# Regulatory non‐coding RNAs in acute myocardial infarction

**DOI:** 10.1111/jcmm.13032

**Published:** 2016-11-23

**Authors:** Yuan Guo, Fei Luo, Qiong Liu, Danyan Xu

**Affiliations:** ^1^ Department of Cardiovascular MedicineThe Second Xiangya HospitalCentral South UniversityChangshaHunanChina

**Keywords:** acute myocardial infarction, microRNAs, long non‐coding RNAs, circular RNAs, apoptosis, inflammation, angiogenesis, fibrosis

## Abstract

Acute myocardial infarction (AMI) is one of the most common cardiovascular diseases that leads to high mortality and morbidity globally. Various therapeutic targets for AMI have been investigated in recent years, including the non‐coding RNAs (ncRNAs). NcRNAs, a class of RNA molecules that typically do not code proteins, are divided into several subgroups. Among them, microRNAs (miRNAs) are widely studied for their modulation of several pathological aspects of AMI, including cardiomyocyte apoptosis, inflammation, angiogenesis and fibrosis. It has emerged that long ncRNAs (lncRNAs) and circular RNAs (circRNAs) also regulate these processes *via* interesting mechanisms. However, the regulatory functions of ncRNAs in AMI and their underlying functional mechanisms have not been systematically described. In this review, we summarize the recent findings involving ncRNA actions in AMI and briefly describe the novel mechanisms of these ncRNAs, highlighting their potential application as therapeutic targets in AMI.

## Introduction

AMI is the most severe cardiovascular event, resulting in high morbidity and mortality worldwide. Atherosclerosis‐induced coronary artery luminal occlusion and plaque rupture is the most common cause of AMI, which is characterized by endothelial injury, lipid accumulation and the formation of atherosclerotic plaque. Cardiomyocyte necrosis and apoptosis with subsequent excessive inflammation are the main causes of myocyte injury and loss in the pathological process of AMI 1. Yet, angiogenesis in the ischaemic area promotes cardiomyocyte survival 2. Eventually, the extent of infarcted ventricular remodelling by fibrosis determines cardiac function and prognosis. Therefore, approaches that inhibit cell death and ventricular fibrosis, regulate inappropriate inflammatory response and promote angiogenesis after AMI are promising therapeutic approaches for improving the prognosis of patients with AMI.

Regulatory ncRNAs are a class of RNA molecules that typically do not code proteins but that functionally regulate protein expression 3. It has been suggested that as much as 98% of the human genome encodes non‐coding transcripts 4. NcRNAs are classified into subgroups according to their transcript length and include small, medium length and lncRNAs. It has been speculated that these non‐coding transcripts are emerging key regulators of gene expression under physiological and pathological conditions. Moreover, there are emerging data that ncRNAs are of crucial importance in cardiovascular diseases, particularly AMI. The AMI‐related ncRNAs that are often studied are miRNAs; lncRNAs and circRNAs, emerging regulatory factors of the pathophysiological processes of AMI. More interestingly, cross‐regulatory networks between miRNAs and lncRNAs/circRNAs were recently identified, endowing these non‐coding transcripts with more potential and comprehensive functions 5.

MiRNAs, the most widely studied small ncRNAs, are about 18–22 nucleotides long and regulate gene expression at post‐transcriptional level through transcript degradation or translational repression 6. Previous studies have revealed critical roles of miRNAs as regulators of the growth, development, function and stress responsiveness of the heart, providing potential therapeutic targets for heart disease 7. MiRNAs are also considered critical regulators for a diverse range of biological processes, including apoptosis, fibrosis, inflammation, angiogenesis and repair in infarcted hearts 8. Therefore, regulating the levels of certain miRNAs after AMI may be helpful for limiting tissue injury, promoting neovascularization and controlling ventricular remodelling, subsequently improving long‐term prognosis.

LncRNAs are a diverse class of heterogeneous transcribed RNA molecules ranging from 200 to 100,000 nucleotides in length. As a newly identified ncRNA in function, the common feature of lncRNAs is that they do not act as vehicles for protein translation 9. Similarly, lncRNAs function as regulators of protein expression. As lncRNAs are also essential for correct and timely regulation of protein expression, they are not only considered to perform functions during the development of an organism, but also play roles in various physiological and pathological conditions, including AMI, even though very little is known of them at present. Furthermore, as lncRNAs are more likely to be expressed in a tissue‐/cell type‐specific manner and can bind to miRNAs to communicate with other RNA targets, lncRNAs have garnered much research attention 10.

CircRNAs, newly discovered endogenous ncRNAs in function, are involved in an area of much research activity because they lack an open end, preventing conventional RNA degradation pathways and acting as more stable RNA molecules 11, 12. CircRNAs are also expressed in a manner of specific to tissue and developmental stage. Similarly, as potential gene regulators, circRNAs modulate many disorders. To date, the function of circRNAs in disease processes has seldom been studied; only a few circRNAs have been proven to play or potentially play cardioprotective roles by acting as molecular sponges targeting miRNAs. Additional exploration of circRNA function will further describe an emerging new factor in the pathological processes of AMI.

Previous studies have reported the importance of both miRNAs and lncRNAs in regulating the pathological processes of AMI; the newly identified functional non‐coding transcripts, circRNAs, have emerged as modulators of AMI. However, the most recently identified ncRNAs and their communication in AMI have not been comprehensively reviewed. Accordingly, we have summarized these ncRNAs and their implicated interactions in modulating cardiomyocyte apoptosis, inflammation, angiogenesis and fibrosis after the acute setting to gain insight into their therapeutic potential in AMI.

## NcRNAs mediate cardiomyocyte apoptosis

**Figure 1:**
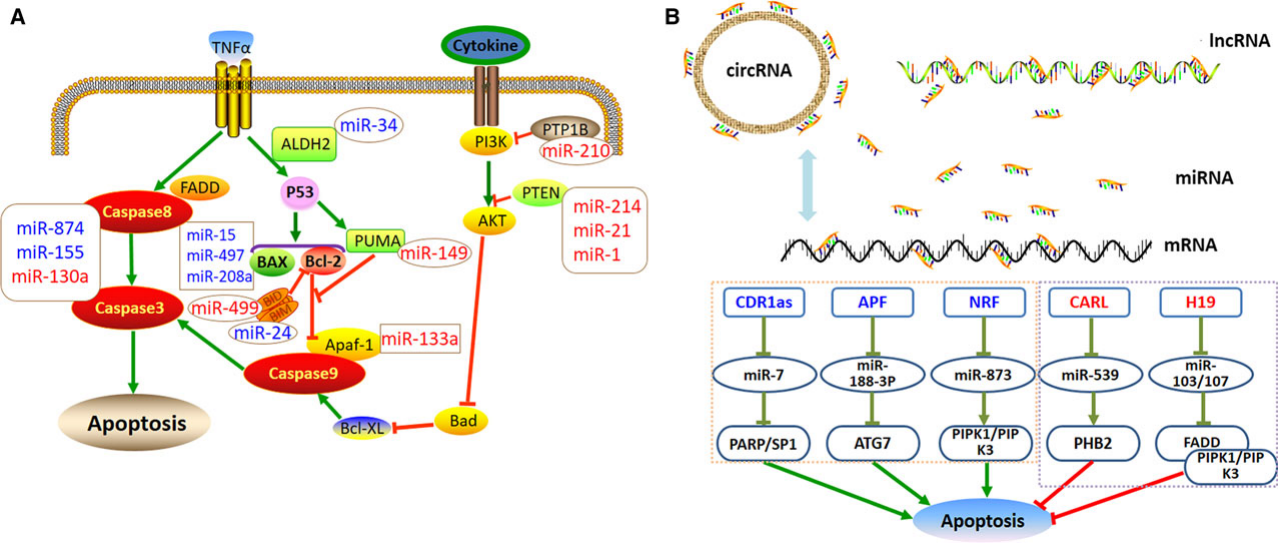
NcRNAs regulate cardiomyocyte apoptosis after acute myocardial infarction. (**A**) MiRNAs mediate cardiomyocyte apoptosis. Pro‐apoptotic miRNAs are marked in blue including miR‐15, miR‐497, miR‐208a, miR‐34a, miR‐24, miR‐874 and miR‐155. Anti‐apoptotic miRNAs are marked in red including miR‐210, miR‐214, miR‐1, miR‐21, miR‐149, miR‐133a, miR‐499 and miR‐130a. (**B**) LncRNAs/circRNAs interact with miRNAs to modulate cardiomyocyte apoptosis. Pro‐apoptotic lncRNAs/circRNAs are marked in blue including APF, NRF and CDR1as. Anti‐apoptotic lncRNAs are marked in red including CARL and H19. APF, autophagy‐promoting factor; ATG7, autophagy‐related protein 7; ALDH2, aldehyde dehydrogenase 2; Apaf‐1, apoptotic protease‐activating factor‐1; Bcl‐2, B‐cell lymphoma 2; BAX, Bcl‐2‐associated X protein; BIM, BCL2‐like 11 apoptosis facilitator; CARL, cardiac apoptosis‐related lncRNA; CDR1as, cerebellar degeneration‐related protein 1 transcript; FADD, Fas‐associated protein with death domain; NRF, necrosis‐related factor; PI3K, phosphoinositide‐3‐kinase; PTP1B, protein tyrosine phosphatase‐1B; PTEN, phosphatase and tensin homolog; PARP, pro‐apoptotic gene poly ADP‐ribose polymerase; PHB2, prohibitin‐2; RIPK1, receptor‐interacting serine/threonine protein kinase 1; RIPK3, receptor‐interacting serine/threonine protein kinase 3; TNF‐α, tumour necrosis factor‐α.

Cardiomyocyte necrosis is a key cellular event in infarct cardiomyopathy, which is generally viewed as an uncontrolled process in AMI. Apoptosis is a highly regulated process that is activated *via* death receptors in the plasma membrane and that mainly occurs in ischaemic areas. The mitochondria‐dependent apoptotic pathway is closely controlled by the ratio of B‐cell lymphoma 2 (Bcl‐2) and the pro‐apoptotic effector Bcl‐2‐associated X protein (BAX). When the pro‐apoptotic effector disrupts the mitochondrial membrane, apoptosis effector caspases are activated and execute apoptosis. Furthermore, extrinsic receptor‐mediated apoptosis is engaged when certain death receptor ligands, such as FAS ligand and tumour necrosis factor‐α (TNF‐α), bind their death receptors on the plasma membrane. Then, caspase‐8 is activated in a manner Fas‐associated protein with death domain (FADD)‐dependent manner. The effector caspases converge on these two pathways. The pro‐apoptotic pathway is counteracted by a series of anti‐apoptotic mediators, including the phosphoinositide‐3‐kinase (PI3K)/AKT pathway. Numerous miRNAs regulate cardiomyocyte apoptosis (Fig. 1A).

### Non‐beneficial miRNAs in cardiomyocyte apoptosis

Some non‐beneficial miRNAs decrease the Bcl‐2/BAX ratio to promote apoptosis. MiR‐15a and miR‐15b are up‐regulated in response to cardiac ischaemia/reperfusion injury and are involved in myocardial apoptosis by targeting Bcl‐2 and the caspase signalling pathway 13. By contrast, miR‐15 inhibition is protective against cardiac injury after MI 14. Forced expression of miR‐497 induced apoptosis in neonatal rat cardiomyocytes, but silencing miR‐497 using a miR‐497 sponge significantly reduced apoptosis; this process was also involved in reducing the expression of the anti‐apoptosis gene *BCL‐2* 15.

Similarly, miR‐24 increases cardiovascular apoptosis in the infarcted myocardium 16. In mice, local adenovirus‐mediated overexpression of miR‐24 increased the percentage of apoptotic cardiomyocyte nuclei by 2.2‐fold. In addition, miR‐24 exerts its pro‐apoptotic function by targeting the pro‐apoptotic gene BCL2‐like 11 apoptosis facilitator (*BIM*) that in turn represses Bcl‐2 expression 17. MiR‐208a also has pro‐apoptotic effects on ischaemic cardiomyocytes, which are related to the increased expression of the pro‐apoptosis gene *BAX* in ischaemic cardiomyocytes 18. MiR‐34a has also been confirmed as an important pro‐apoptosis regulator in AMI 19; it is increased after ischaemia and exerts its pro‐apoptosis function by negatively regulating the anti‐apoptotic protein aldehyde dehydrogenase‐2 (ALDH2), which also decreases the Bcl‐2/BAX ratio 20.

Several other miRNAs promote cardiomyocyte apoptosis after AMI by directly targeting the caspase family. Wang *et al*. 21 demonstrated that miR‐874 is involved in H_2_O_2_‐induced cardiomyocyte death by increasing caspase‐8 after MI. The authors further confirmed the apoptosis‐promoting function of miR‐874 using a miR‐874 antagomir that significantly attenuated H_2_O_2_‐induced cell death. MiR‐155 deficiency prevented ischaemia/reperfusion injury‐induced apoptosis in an AMI mouse model 15. Furthermore, Eisenhardt *et al*. 22 found that miR‐155 aggravated apoptosis post‐AMI by increasing the expression of the apoptosis‐related caspase‐3.

MiR‐92a promoted apoptosis in the heart after MI, and treatment with antagomiR‐92a to inhibit miR‐92a *in vivo* reversed this process. Unfortunately, the antagomiR‐92a‐induced reduction in cardiomyocyte apoptosis was not observed *in vitro*, suggesting that an indirect mechanism mediates the anti‐apoptotic activity of antagomiR‐92a *in vivo* 8. Overall, inhibiting these miRNAs may be new therapeutic approaches in AMI.

### Protective miRNAs in cardiomyocyte apoptosis

The PI3K/AKT pathway is the main signalling pathway for inhibiting apoptosis that is consistently activated with activation of the pro‐apoptotic pathway after AMI. Several miRNAs protect cardiomyocytes against apoptosis after AMI by activating PI3K and its downstream regulators. MiR‐210 inhibited apoptosis in mice after MI, and miR‐210 overexpression prevented cardiomyocyte apoptosis by down‐regulating protein tyrosine phosphatase‐1B that subsequently activated the PI3K/AKT pathway 23. MiR‐214 is a newly identified miRNA that inhibits cardiomyocyte apoptosis. Overexpression of miR‐214 in an AMI rat model decreased the size of the infarcted area, improved heart function and haemodynamic status and inhibited left ventricular remodelling. The miR‐214‐mediated protective mechanism is based on the repression of phosphatase and tensin homolog (PTEN), which acts as a PI3K inhibitor 24. In an AMI mouse model, overexpressing miR‐1 in embryonic stem cells and transplanting them into infarcted myocardium inhibited cardiomyocyte apoptosis and improved cardiac function after 4‐week treatment, which was related to reduced PTEN levels and caspase‐3 activity 25, 26. Similarly, miR‐21 was involved in trimetazidine‐induced anti‐apoptosis during ischaemia/reperfusion injury, and increased miR‐21 expression inhibited cardiomyocyte apoptosis 27. Forced expression of miR‐21 up‐regulated PI3K/AKT activity by suppressing PTEN expression and increasing the Bcl‐2/BAX ratio, which in turn reduced caspase‐3 expression and finally counteracted the apoptotic effect 28.

Another mechanism of the miR‐21‐induced anti‐apoptosis effect in H_2_O_2_‐mediated cardiomyocytes is directly inhibiting pro‐apoptotic protein programmed cell death 4 (PDCD4) expression 29, 30, 31. Cardiac‐specific miR‐499 was widely reported as an important biomarker reflecting myocardial damage in AMI 32, 33. Overexpression of miR‐499 favoured cardiomyocyte survival and inhibited apoptosis. More interestingly, it was recently reported that miR‐499 protects cardiomyocytes from H_2_O_2_‐induced apoptosis and rat AMI models by suppressing expression of the pro‐apoptotic protein PDCD4 and phosphofurin acidic cluster sorting protein 2, thereby blocking *Bid* expression and BID mitochondrial translocation 34, 35.

Other miRNAs also directly target the caspase family. Dakhlallah *et al*. 36 transfected mesenchymal stem cells with miR‐133a and found that it prevented apoptosis by directly targeting apoptotic protease‐activating factor‐1, which down‐regulated caspase‐9 and caspase‐3 expression. Lu *et al*. 37 found that MI induced myocardial apoptosis and increased caspase‐3/7 and caspase‐8 activity by 105.6% and 71.3%, respectively, when compared with sham controls, while lentivirus transfection of miR‐130a overexpression markedly reduced caspase‐3/7 and caspase‐8 activity by 22.9% and 30.8%, respectively, as compared with the controls. Ding *et al*. 38 reported that miR‐149 contributed to inhibition of apoptosis after MI by regulating the pro‐apoptotic protein PUMA, which in turn activated caspase‐9 to promote apoptosis. Hence, activating these miRNAs is a promising target for treating AMI.

### LncRNAs and circRNAs in cardiomyocyte apoptosis

Recently, some lncRNAs and circRNAs were identified as vital biomarkers and promising therapeutic targets in AMI 39, 40 (Table 1 and Fig. 1B). The circulating lncRNA urothelial carcinoma‐associated 1 (UCA1) was down‐regulated within 3 days after the onset of AMI 41. Microarray analysis of MI mice showed that two lncRNAs, MI‐associated transcript 1 and 2, were significantly up‐regulated fivefold and 13‐fold, respectively, after MI 42. Moreover, myosin heavy chain‐associated RNA transcripts (MHRT), a heart‐specific lncRNA, were significantly elevated in the blood of patients with AMI as compared with healthy controls (*P* < 0.05). In a H_2_O_2_‐induced neonatal rat cardiac myocyte injury model, MHRT was also up‐regulated in injured cardiac myocytes, and short interfering RNA knock‐down of the *Mhrt* gene led to more apoptotic cells than in the non‐target control (*P* < 0.01), indicating that MHRT is not only a biomarker of ischaemic cardiomyocytes but also a protective lncRNA for cardiomyocytes and a promising therapeutic target of AMI 43.

**Table 1:** Long non‐coding RNAs as biomarkers in acute myocardial infarction

	LncRNAs	Regulation after AMI	Relation to other biomarkers
Yan *et al*. 41	UCA1	Down‐regulation	Inversely related to miR‐1 level
Zangrando *et al*. 42	MIRT1/MIRT2	Up‐regulation	Negatively correlated with infarct size; positively correlated with EF value
Zhang *et al*. 43	MHRT	Up‐regulation	Inversely related to cardiomyocyte apoptosis
Vausort *et al*. 56	ANRIL	Down‐regulation	Positively correlated to lymphocytes and monocytes; negatively related to MMP9, WBC, neutrophils, platelets
Vausort *et al*. 56	MIAT	Down‐regulation	Positively related to lymphocytes; negatively related to neutrophils, platelets
Vausort *et al*. 56	MALAT1	Up‐regulation	Negatively related to platelets
Vausort *et al*. 56	aHIF	Up‐regulation	Positively related to WBC, neutrophils CRP, MMP9, TIMP1; negatively related to lymphocytes
Qu *et al*. 80	NONMMUT022554	Up‐regulation	Positively correlated with fibrosis gene expression

ANRIL, cyclin‐dependent kinase inhibitor 2B antisense RNA 1; aHIF, hypoxia‐inducible factor 1A antisense RNA 2; CRP, C‐reactive protein; EF, ejection fraction; MIRT1, MI‐associated transcript 1; MIRT2, MI‐associated transcript 2; MIAT, myocardial infarction‐associated transcript; MALAT1. metastasis‐associated lung adenocarcinoma transcript 1; MMP9, matrix metalloproteinase 9; TIMP1, tissue inhibitor of metalloproteinase 1; UCA1, urothelial carcinoma‐associated 1; WBC, white blood cell.

In addition, other lncRNAs interact with miRNAs to exert their apoptotic inhibitory function. MiR‐188‐3p inhibited autophagy under pathological conditions by targeting autophagy‐related protein 7. Wang *et al*. 44 found that the lncRNA autophagy‐promoting factor regulated autophagic cell death by down‐regulating miR‐188‐3p, thereby promoting autophagy after MI. Cardiac apoptosis‐related lncRNA (CARL) suppressed mitochondrial fission and apoptosis by decreasing endogenous miR‐539 levels by acting as a sponge, which in turn up‐regulated prohibitin 2 expression to inhibit apoptosis 45. MiRNA‐103/107 and the lncRNA H19 also mediate cardiomyocyte survival after AMI. H19 bound directly to miR‐103/107, suppressing receptor‐interacting serine/threonine protein kinase (RIPK)1/RIPK3 and FADD‐dependent death in foetal cardiomyocyte‐derived H9C2 cells and in an MI mice model 46. The lncRNA necrosis‐related factor (NRF) targets miR‐873 and RIPK1/RIPK3 to regulate cardiomyocyte death. An endogenous sponge RNA, NRF repressed miR‐873 expression, which in turn increased RIPK1/RIPK3 and cardiomyocyte death 47. Thus, these lncRNAs are potential therapeutic targets of AMI by inhibiting cardiomyocyte death.

The circRNA cerebellar degeneration‐related protein 1 transcript (CDR1as) has 63 conserved binding sites for miR‐7, by which CDR1as could function as an miR‐7 sponge to regulate post‐transcriptional gene expression 48. Intriguingly, Geng *et al*. 49 recently reported the CDR1as/miR‐7a pathway in cardiomyocytes and explored the underlying function of miR‐7a in protection against AMI. They found that CDR1as and miR‐7a were both up‐regulated in MI mice or cardiomyocytes under hypoxia treatment. However, overexpression of CDR1as *in vivo* increased cardiac infarct size, while miR‐7a overexpression reversed these changes. Furthermore, CDR1as functioned as a powerful miR‐7a sponge in myocardial cells, and miR‐7a protected cardiomyocytes from injury after MI by inhibiting the expression of the pro‐apoptotic gene poly ADP‐ribose polymerase (*PARP*) and *SP1*. This indicates that CDR1as may be a promising anti‐apoptosis target.

## NcRNAs regulate inflammation around infarct areas

Cardiac cell death after ischaemia subsequently induces inflammatory cascades. Proper inflammatory reaction helps to clear cellular debris and trigger repair mechanisms after AMI, while excessive inflammation is a critical factor in aggravating cardiomyocyte injury and death. Therefore, modulating excessive inflammatory response is essential for preventing cardiomyocyte death 50. Some miRNAs play critical roles in reducing the inflammatory response after AMI (Table 2).

**Table 2:** MicroRNAs regulated inflammation in acute myocardial infarction

	NcRNAs	Function	Targets	Modulation
Liu *et al*. 51	miR‐150	Inhibit inflammation	Inhibit CXCR4	Increase expression
Yao *et al*. 52	miR‐155	Promote inflammation	Modulation T helper cells differentiation	Inhibit expression
Eisenhardt *et al*. 22	miR‐155	Promote inflammation	Increase TNF‐α, IL‐1b, CD105 and leucocyte infiltration	Inhibit expression
Ibrahim *et al*. 54	miR‐146a	Inhibit inflammation	Suppress IRAK1 and TRAF6	Increase expression
Toldo *et al*. 55	miR‐21	Inhibit inflammation	Attenuate the formation of inflammasome	Increase expression

CXCR4, chemokine receptor 4; IL‐1b, interleukin‐1b; IRAK1, interleukin‐1 receptor‐associated kinase 1; TNF‐α, tumour necrosis factor‐α; TRAF6, tumour necrosis factor receptor‐associated factor 6.

The inflammatory response is mainly involved in monocyte cell migration and the production of a cluster of proinflammatory cytokines, which then initiate a cascade reaction. The overexpression of miR‐150 in mice was critical for monocyte migration and proinflammatory cytokine production, resulting in cardioprotective effects against AMI injury. This was related to miR‐150 inhibition of chemokine receptor 4 and subsequently reduced inflammatory Ly‐6C^high^ monocyte invasion after AMI 51. Furthermore, the inflammation‐related miR‐155 was down‐regulated by approximately 60% in patients with acute coronary syndrome, which was consistent with the expression of interleukin‐17A in peripheral blood mononuclear cells, suggesting that it is essential for T helper cell differentiation 52. A similar study reported that, in an AMI mouse model, miR‐155 significantly increased TNF‐α, IL‐1b and CD105 expression and leucocyte infiltration after AMI, and that miR‐155 deficiency prevented ischaemia/reperfusion injury‐induced tissue necrosis and attenuated inflammatory cell infiltration 22.

There is evidence that the inflammation‐related miR‐146a and miR‐21 are increased by approximately twofold in patients with acute coronary syndrome 52. Liu *et al*. 53 found that miR‐146a and miR‐21 were positively related with MI, which was consistent with the C‐reactive protein levels and leucocyte counts, indicating that the two miRNAs are involved in post‐AMI inflammation. Ibrahim *et al*. 54 reported post‐AMI inflammation in miR‐146a‐enriched exosomes and that miR‐146a performed its function by suppressing IL‐1 receptor‐associated kinase 1 and TNF receptor‐associated factor 6 expression. Toldo *et al*. 55 found that exogenous hydrogen sulphide reduced myocardial ischaemia and inflammation in cardiomyocytes after MI by attenuating the formation of inflammasomes in a miR‐21‐dependent manner. These inflammatory miRNAs might be potent therapeutic targets in the setting of ischaemic heart disease.

Recent findings have also determined that several lncRNAs act as inflammatory biomarkers after AMI (Table 1). There was a positive association between cyclin‐dependent kinase inhibitor 2B antisense RNA 1 and the percentage of lymphocytes and monocytes, but it was inversely associated with white blood cell count, neutrophil count, platelet count and matrix metalloproteinase 9 (MMP9). MI‐associated transcript (MIAT) was positively associated with lymphocyte count and negatively associated with neutrophil and platelet counts. Metastasis‐associated lung adenocarcinoma transcript 1 was negatively associated with platelet count. Hypoxia‐inducible factor 1A antisense RNA 2 was also positively associated with white blood cell count, neutrophil count, C‐reactive protein, MMP9 and tissue inhibitor of metalloproteinase‐1 56. These findings suggest that these lncRNAs play important roles in AMI.

## NcRNAs modulate angiogenesis in ischaemic areas

**Figure 2:**
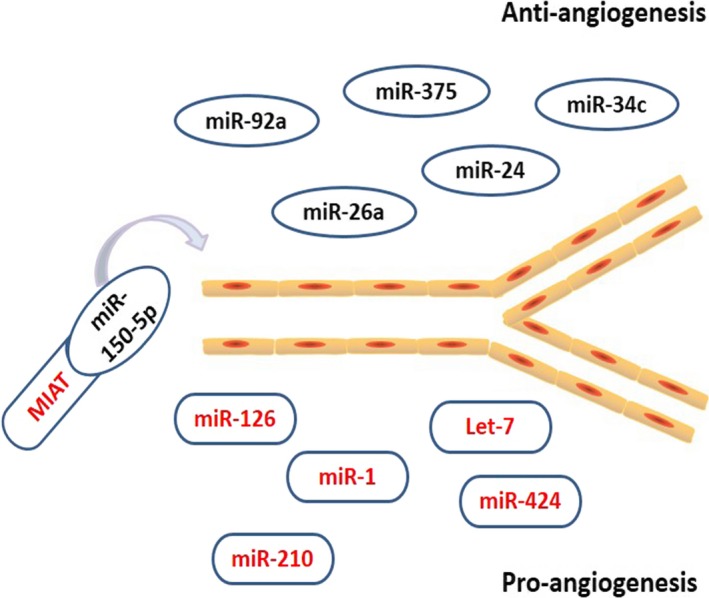
NcRNAs mediate angiogenesis around infarct areas after acute myocardial infarction. Anti‐angiogenesis ncRNAs are marked in black including miR‐92a, miR‐26a, miR‐24, miR‐34c, miR‐375 and miR‐150‐5p. Pro‐angiogenesis ncRNAs are marked in red including miR‐126, miR‐1, miR‐210, miR‐424, Let‐7 and MIAT. MIAT, myocardial infarction‐associated transcript.

Angiogenesis is a critical component in post‐AMI early tissue repair that also participates in limiting the infarct size and reducing myocardial apoptosis. MiRNAs also modulate angiogenesis (Fig. 2 and Table 3). MiR‐92a is the most widely studied miRNA for inhibiting angiogenesis after AMI. Bellera *et al*. 57 demonstrated that a single intracoronary administration of antagomiR‐92a encapsulated in specific microspheres inhibited miR‐92a, resulting in significant vessel growth in a local, selective and sustained manner in a pig model of AMI. Bonauer *et al*. 8 reported that miR‐92a controlled blood vessel growth in an MI mouse model by decreasing integrin α5; systemic administration of antagomir to inhibit miR‐92a enhanced blood vessel growth and functional recovery of damaged tissue. Similarly, Hinkel *et al*. 58 found that local delivery of locked nucleic acid (LNA)‐modified antisense miR‐92a directed against miR‐92a expression significantly reduced infarct size and improved the recovery of cardiac function in pigs after MI by targeting integrin α5. Consequently, miR‐92a may serve as a valuable therapeutic target in the ischaemic disease setting.

**Table 3:** Non‐coding RNAs regulated angiogenesis in acute myocardial infarction

	NcRNAs	Function	Targets	Modulation
Bonauer *et al*. 8	miR‐92a	Inhibit angiogenesis	Decrease ITGA5	Inhibit expression
Hinkel *et al*. 58	miR‐92a	Inhibit angiogenesis	Decrease ITGA5	Inhibit expression
Icli *et al*. 59	miR‐26a	Inhibit angiogenesis	Inhibit BMP/SMAD1	Inhibit expression
Meloni *et al*. 17	miR‐24	Inhibit angiogenesis	Decrease eNOS, increase PAK4, GATA2	Inhibit expression
Kang *et al*. 60	miR‐34c	Inhibit angiogenesis	Decrease SCF increase KLF4, PAI‐1	Inhibit expression
Garikipati *et al*. 61	miR‐375	Inhibit angiogenesis	Negatively regulate PDK‐1	Inhibit expression
Huang *et al*. 63	miR‐126	Promote angiogenesis	Up‐regulation of VEGF, bFGF and DLL‐4	Increase expression
Wang *et al*. 64	miR‐126	Promote angiogenesis	Promoting VEGF, FGF repressing SPRED1	Increase expression
van Mil *et al*. 65	miR‐1	Promote angiogenesis	Inhibit SPRED1	Increase expression
Chen *et al*. 67	Let‐7	Promote angiogenesis	Suppress AGO1, increase VEGF produce	Increase expression
Hu *et al*. 23	miR‐210	Promote angiogenesis	Release angiogenic factors; decrease EFNA3 expression	Increase expression
Ghosh *et al*. 68	miR‐424	Promote angiogenesis	stabilize HIF‐α	Increase expression
Yan *et al*. 69 \	MIAT	Promote angiogenesis	Decrease miR‐150‐5p Increase VEGF	Increase expression

AGO1, argonaut 1; BMP, bone morphogenic proteins; bFGF, basic fibroblast growth factor; DLL‐4, notch ligand Delta‐like 4; eNOS, endothelial nitric oxide synthase; GATA2, globin transcription factor binding protein 2; HIF‐α, hypoxia‐inducible factor‐α; ITGA5, integrin α5; KLF4, Krüppel‐like factor 4; MIAT, myocardial infarction‐associated transcript; PAK4, pro‐angiogenic p21 protein‐Cdc42/Rac‐activated kinase 4; PAI‐1, plasminogen activator inhibitor‐1; PDK‐1, 3‐phosphoinositide‐dependent protein kinase‐1; SPRED1, sprouty‐related EVH1 domain‐containing protein 1; SCF, stem cell factor; VEGF, vascular endothelial growth factor.

Several other miRNAs, including miR‐26a, miR‐24, miR‐34c and miR‐375, are involved in suppressing angiogenesis in AMI. MiR‐26a overexpression in an AMI mouse model and in human subjects with acute coronary syndrome attenuated angiogenesis. By contrast, miR‐26a inhibitor induced angiogenesis by inhibiting bone morphogenic protein (BMP)/SMAD1 signalling, thereby reducing myocardial infarct size 59. Moreover, direct antagomirs against miR‐24 or local adenovirus‐mediated miR‐24 decoy delivery improved recovery after AMI in mice. MiR‐24 inhibition increased blood vessels in infarcted myocardium by increasing endothelial nitric oxide synthase (eNOS) and decreasing the pro‐angiogenic p21 protein‐Cdc42/Rac‐activated kinase 4 (PAK4) and globin transcription factor binding protein 2 (GATA2) 16, 17. High glucose‐induced miR‐34c expression impaired angiogenic activity after MI by reducing stem cell factor (SCF) and increasing Krüppel‐like factor 4 (KLF4) and plasminogen activator inhibitor‐1 (PAI‐1) 60. MiR‐375 also inhibits angiogenesis after MI; IL‐10‐induced miR‐375 decrease exerted a pro‐angiogenic effect by up‐regulating 3‐phosphoinositide‐dependent protein kinase‐1 (PDK‐1) 61. Accordingly, inhibiting these harmful miRNAs after AMI may be a promising therapeutic target and may improve the prognosis after acute settings.

However, a cluster of miRNAs increases angiogenesis after AMI. MiR‐126 is considered a positive regulator of angiogenesis after AMI 62. Huang *et al*. 63 reported that miR‐126 overexpression up‐regulated vascular endothelial growth factor (VEGF), basic fibroblast growth factor (FGF) and notch ligand Delta‐like 4 (DLL4) in mesenchymal stem cells and thereby enhanced functional angiogenesis in the ischaemic myocardium. Wang *et al*. 64 determined that the pro‐angiogenic actions of miR‐126 after MI were related to repressed expression of sprouty‐related EVH1 domain‐containing protein 1 (SPRED1), an intracellular inhibitor of angiogenic signalling. Furthermore, miR‐1 also enhanced the angiogenic effects of progenitor cells by inhibiting SPRED1 expression 65.

Other miRNAs have been identified to promote angiogenesis after AMI. Let‐7 plays an active role in the pathogenesis of MI 66. Chen *et al*. 67 reported that hypoxia induced let‐7 expression, which suppressed Argonaut 1 (AGO1) and increased VEGF to promote angiogenesis. Using an MI mouse model, Hu *et al*. 23 demonstrated that miR‐210 improved angiogenesis by releasing angiogenic factors; overexpressing miR‐210 resulted in the down‐regulation of the anti‐angiogenic gene *EFNA3* and promoted angiogenesis. Ghosh *et al*. 68 reported that miR‐322/424 was up‐regulated after MI and hypoxia, and increased miR‐424 targeted cullin‐2 to stabilize hypoxia‐inducible factor α isoforms and promote angiogenesis. Accordingly, enhancing the expression of the pro‐angiogenic miRNAs might be a valuable therapeutic target in AMI.

Research has seldom discussed the roles of lncRNAs in angiogenesis after AMI. Previous findings have only reported that MIAT is related to angiogenesis, functioning as a competing endogenous RNA by sponging miR‐150‐5p in retinal endothelial cells to regulate VEGF levels. That is, MIAT overexpression acted as a sink for miR‐150‐5p, which in turn increased VEGF levels and promoted angiogenesis 69. Further exploration of more lncRNAs involved in angiogenesis may determine whether they are potent factors that promote angiogenesis after AMI.

## NcRNAs regulate fibrosis in infarct regions

**Figure 3:**
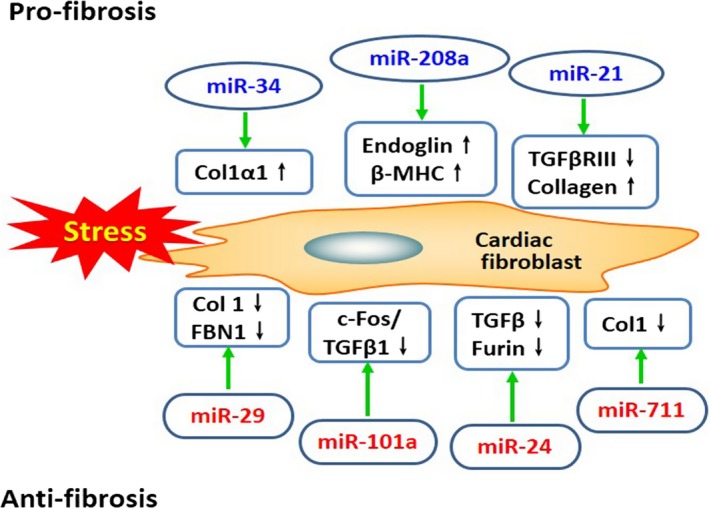
MiRNAs modulate cardiac fibrosis in infarct regions after acute myocardial infarction. Pro‐fibrosis miRNAs are marked in blue including miR‐34, miR‐208a and miR‐21. Anti‐fibrosis miRNAs are marked in red including miR‐29, miR‐101a, miR‐24 and miR‐711. Col1: collagen 1, Col1a1: collagen 1a1, β‐MHC: β‐myosin heavy chain, FBN1: fibrillin 1, TGFβ: transforming growth factor beta, TGFβRIII: transforming growth factor beta receptor III.

Cardiac fibroblasts are activated and subsequently produce excessive extracellular matrix (ECM) proteins after MI, which ultimately impairs cardiac function and leads to interstitial fibrosis and remodelling of the heart. Accordingly, inhibiting excessive ECM secretion and deposition is an important therapeutic strategy for improving the prognosis of AMI. Several miRNAs have been implicated in the pathology of cardiac fibrosis after AMI (Fig. 3). Inhibiting the miR‐34 family improved cardiac function in mice and attenuated pathological remodelling after MI. Bernardo *et al*. 70 reported that silencing entire miR‐34 family protected the heart against pathological cardiac remodelling and improved cardiac function. The authors also found elevated collagen (Col) 1α1 gene expression in the infarct zone after MI in mice. However, MI mice treated with LNA‐anti‐miR‐34 trended towards lower Col1α1 expression. Huang *et al*. 71 further demonstrated that inhibiting miR‐34a reduced the severity of experimental cardiac fibrosis in mice after AMI, indicating that miR‐34a plays a critical role in the progression of cardiac tissue fibrosis by directly inducing pro‐fibrotic pathway transforming growth factor beta 1 (TGFβ1)/Smad4.

Furthermore, miR‐208a and miR‐21 were also involved in promoting cardiac fibrosis after MI. MiR‐208a was increased in rats with AMI, which significantly increased the area of myocardial fibrosis compared with the sham group. It was further believed that the cardiac fibrosis action induced by miR‐208a was related to endoglin activation and β‐myosin heavy chain expression 72. MiR‐21 is an important regulatory molecule in the pathogenic process of myocardial fibrosis after MI. MiR‐21 was up‐regulated in the border zone of the infarcted region after AMI in mice, which could increase the collagen content and lead to cardiac fibrosis, which was partially related to inhibition of transforming growth factor beta receptor III (TGFβRIII) expression 73. Therefore, inhibiting these miRNAs may be a promising strategy for treating cardiac fibrosis after AMI.

However, some miRNAs inhibit cardiac fibrosis after AMI, and miR‐29 is the most well‐studied anti‐fibrosis miRNA. van Rooij *et al*. 74 showed that miR‐29 expression was down‐regulated after MI, thereby inducing collagen overexpression and cardiac fibrosis. They also reported that the mechanism of collagen overexpression induced by low miR‐29 levels was related to up‐regulation of its targets Col1α1, Col1α2, Col1α3 and fibrillin 1 in the infarcted region. Melo *et al*. 75 also reported that swimming training improved ventricular function after MI in rats by improving cardiac miR‐29a and miR‐29c levels, thereby preventing COLIAI and COLIIIAI expression in the border region and remote myocardium of the infarcted left ventricle.

Additionally, several other miRNAs, including miR‐101a, miR‐24 and miR‐711, also counteract cardiac fibrosis and attenuate the remodelling process after MI. MiR‐101a is a novel identified anti‐fibrotic miRNA that suppresses cardiac fibrosis and improves the impaired cardiac function in post‐infarct rats, which involves the underlying mechanism of inhibiting the c‐Fos/TGFβ1 and TGFβR1 pathways 76, 77. MiR‐24 was also down‐regulated in mouse heart after MI. MiR‐24 improved heart function and attenuated fibrosis in the infarct border zone of the heart 2 weeks after MI induced through intramyocardial injection of lentiviruses, which was related to the regulation of furin (a protease that controls latent TGFβ activation) and reduced TGFβ (a pathological mediator of fibrotic disease) secretion and Smad2/3 phosphorylation in cardiac fibroblasts 78. Moreover, up‐regulating miR‐711 inhibited cardiac fibrosis in rats with MI, which was mainly related to the reduced COLI levels 79. Therefore, these miRNAs act as regulators of cardiac fibrosis and represent potential therapeutic targets of tissue fibrosis after AMI.

Recently, lncRNAs were also studied in cardiac fibrosis after AMI. A recent study detected lncRNAs variation in mice 4 weeks after MI and found that at the peri‐infarct region, 53 lncRNAs had been up‐regulated by more than twofold and 37 lncRNAs has been down‐regulated by over 0.5‐fold. Meanwhile, NONMMUT022554 was identified as the most significantly up‐regulated lncRNA and was positively correlated with six up‐regulated genes involved in ECM‐receptor interactions 80. Furthermore, some lncRNAs were also related to cardiac fibrosis. For example, overexpression of H19 contributed to cardiac fibroblast proliferation and fibrosis 81. LncRNA cardiac hypertrophy‐related factor also regulates cardiac hypertrophy 82. However, the pro‐fibrotic function of these lncRNAs in MI has not been identified, and with further exploration, they may also be potential targets for treating AMI.

## LncRNA/circRNA–miRNA‐mediated interaction

Generally, miRNAs bind directly to their target mRNAs by complementary base pairing and trigger mRNA cleavage based on the degree of complementarity. MiRNAs regulate gene expression, mostly at the 3′ untranslated region, thereby decreasing mRNA translation and stability 83. LncRNAs and circRNAs function as molecular regulators by determining gene expression from transcription to translation. More interestingly, lncRNAs and circRNAs both contain complementary binding sites to miRNAs and act as endogenous miRNA sponges; miRNAs in turn interact with mRNAs, serving as negative regulators of protein expression. Moreover, the regulatory mechanism of lncRNAs and circRNAs mainly focuses on acting as molecular sponges by binding to miRNAs and forming an lncRNA/circRNAs–miRNA axis to regulate the expression of the related mRNAs and proteins 9.

As previously reported, CARL induces cardiac myocyte apoptosis by acting as an endogenous sponge and reducing miR‐539 levels, which subsequently inhibits mitochondrial fission and apoptosis in the heart 45. H19 binds directly to miR‐103/107, repressing RIPK1 and RIPK3, negatively regulating FADD and reducing apoptosis 46. Similarly, MIAT acts as a competing endogenous RNA sponge to miR‐150‐5p, regulating VEGF levels and endothelial cell function 69. The circRNA CDR1as functioned as a miR‐7a sponge in myocardial cells and regulated cardiomyocyte apoptosis after MI 49. These findings demonstrate the critical mechanism in the miRNA regulatory networks, which are involved in the complex, competitive endogenous RNA network.

## Conclusion and clinical perspectives

Recently, novel therapeutic approaches for AMI have received much attention, and numerous potential targets, especially ncRNAs, have been studied. Emerging data suggest that ncRNAs play important roles in several physiological and pathological processes in AMI. The best‐studied ncRNAs are miRNAs; several miRNAs might be attractive candidates for improving recovery by controlling the pathological conditions of AMI. For example, several animal studies have demonstrated that miR‐92a inhibitors reduce cardiomyocyte apoptosis and promote angiogenesis, thereby decreasing infarct size and improving prognosis after AMI. Apart from miRNAs, lncRNAs and circRNAs such as MIAT and CDR1as have emerged as potential regulators of AMI progression. However, these recently identified ncRNAs in AMI and their interactions have not been completely described. We analysed the ncRNAs implicated in the processes of AMI to gain insight into their potential functions as therapeutic targets of AMI.

Although the evidence is convincing and indicates that manipulating ncRNA levels is efficient for reducing infarct area and for restoring left ventricular mass, as well as promoting functional recovery after AMI in animal models, the development of ncRNA therapy faces several challenges. Moreover, it is difficult to use the regulatory ncRNAs in the clinic in a short time. First, our understanding of the biology of ncRNAs is far from complete; this is especially true for lncRNAs and circRNAs, which are involved in more complex and wider functions by interacting with miRNAs. Next, targeting ubiquitously expressed ncRNAs may encounter challenges with respect to off‐target effects and unwanted adverse effects in other cells or tissues. In addition, as gene therapy requires the use of vectors to some extent, the safety and efficacy still requires thorough evaluation. Accordingly, although ncRNAs have promising application prospects, they have not reached the stage that would allow easy clinical translation. In future research, some cases may require cell type‐specific delivery strategies. Preclinical trials (such as the antagomiR anti‐miR‐122 for treating hepatitis C virus infection 84, 85) for evaluating their safety and feasibility will aid the development of ncRNA therapeutics for AMI.

## Conflict of interest

The authors reported no relationships that could be construed as a conflict of interest.
